# Analysis of laboratory critical values at a referral Spanish tertiary university hospital

**DOI:** 10.11613/BM.2019.010704

**Published:** 2018-12-15

**Authors:** Ariadna Arbiol-Roca, Sofía Corral-Comesaña, Ruth Cano-Corres, María José Castro-Castro, Macarena Dastis-Arias, Dolors Dot-Bach

**Affiliations:** 1 Laboratori Clínic, Hospital Universitari de Bellvitge, Hospitalet de Llobregat, Barcelona, Spain; 2 Synlab Diagnósticos Globales SA, Esplugues de Llobregat, Barcelona, Spain; 3 Clinical Laboratory, Biochemistry Department, Parc Taulí Hospital Universitari, Institut d’Investigació i Innovació Parc Taulí I3PT, Universitat Autònoma de Barcelona, Sabadell, Spain

**Keywords:** critical values limits, critical values notification, patient safety

## Abstract

**Introduction:**

The aim of this study was to analyse critical value data from our laboratory and compare our critical value reporting policy with others in the literature.

**Materials and methods:**

Analysis of critical values was performed on data obtained over a 6-month period in a tertiary university hospital.

**Results:**

We identified 5723 critical values, of which approximately 80% came from STAT testing (4577), 15% from routine inpatients testing (884) and 5% from routine outpatients testing (262). The highest proportion of critical values corresponded to oxygen partial pressure (17.7%), followed by potassium ion (17.6%) concentrations. The parameters associated with the highest critical value notification percentage in emergency patients were pH, haematocrit, glucose, potassium ion and haemoglobin concentrations. In inpatients, these parameters were glucose, phosphate, haemoglobin, sodium ion and potassium ion concentrations. In outpatients, they were calcium and potassium concentrations.

**Conclusions:**

The analysis of critical values in our hospital is in accordance with that reported in the literature. Our findings demonstrate the importance of incorporating improvement actions not only in critical value notification, but especially in the registration of this activity.

## Introduction

A critical value is defined as “a laboratory test result that represents a pathophysiologic state at such variance with normal as to be life-threatening unless something is done promptly and for which some corrective action could be taken” (*1*).

The laboratory accrediting agencies have made critical value reporting part of the requirements for accreditation (*2*, *3*). Furthermore, the immediate notification of a critical value as a special requisite has been recognised and implemented worldwide through the International Organization for Standardization (ISO) 15189:2012, and has been adopted as a standard of Good Laboratory Practice (*4*, *5*).

The literature indicates that there is no consensus on the biomarkers which are more likely to result in critical values, nor on what the critical value limits should be. A consensus must be reached among physicians on the list of biomarkers and the critical value limits of each, which should be established by each laboratory (*6*-*9*). Several organizations have published guidelines for the reporting of critical results, one of which is issued by the British Royal College of Pathologists (*10*). Furthermore, independent lists are needed for different study populations, as critical values will differ between neonatal, paediatric and adult care patients (*11*).

Instant communication of these critical values leads to faster diagnostic processes and a rapid change in patient management. Clinical laboratories must establish optimal critical value limits in order to achieve a balance between informing clinical staff and overloading them with results that do not require urgent action (*12*-*14*).

Critical values are usually detected by the laboratory technical staff. It is necessary to ensure the validity of critical values from the beginning to ensure there are no potential sources of interference in order to ensure patient safety (*15*). Once a critical value has been validated, it is necessary to notify the physician. Although electronic patient records, laboratory information system (LIS) and hospital information system (HIS) software have been widely introduced in hospitals, the most common reporting system is still telephone communication directly by laboratory staff or *via* call centres (*16*). However, some laboratories have started employing automated systems (*14*, *17*).

Communication of critical values is an important issue; however, the guidelines do not identify who should be responsible for receiving the critical values notification (head physician, registered nurse, attending physician, *etc.*). This notification should be registered, as well as confirmation that the caregiver has accepted responsibility for follow up. Thus, the aforementioned critical value notification procedure of clinical laboratories must be carefully considered, as it affects patient safety and the efficiency and quality of clinical care (*18*).

In the present study, we analysed 6-month period of critical value data and reporting practices to compare our results with those reported in the literature. The aim of our study was to determine the situation of our laboratory for the notification of critical values in order to detect strengths and weaknesses, which would enable us to improve the whole process.

## Materials and methods

The clinical laboratory of University Hospital of Bellvitge has been accredited by the ISO 15189 since February 2008. Every year, the clinical laboratory performs 3.5 million tests, of which 30% are urgent requests.

This retrospective study, conducted over a 6-month period (January to June 2017), was developed to evaluate the implemented critical value communication process in a Spanish tertiary university hospital. All data was obtained from reports generated from the LIS (Omega3000: Roche Diagnostics SL, Spain). Data were exported from the LIS into Microsoft Excel (Microsoft Office 2003®, Alburquerque, EEUU). The parameters recorded included calcium (II), calcium ion, carbon dioxide partial pressure, carboxyhaemoglobin, erythrocytes, glucose, haemoglobin, oxygen partial pressure, phosphate, pH, potassium ion, sodium ion and thrombocytes.

Laboratory staff is notified of the presence of a critical value by the LIS, which is configured in such a way that a red warning flag automatically shows up when a critical value appears. The result is then checked for analytical reliability, and once the potential margin for error has been eliminated and the result validated, the person responsible for the patient’s healthcare is notified by the laboratory technician. With regard to critical values, our laboratory has established two separate processes for inpatients and outpatients. For inpatients, either the nurse or the medical department are notified of critical values by phone. For outpatients, however, the laboratory staff gets in touch *via* the hospital’s call centre, who call the attending physician as well as send them an email. The call centre then sends confirmation to the laboratory that the above has taken place. All critical values reported for both inpatients and outpatients must be collected and registered in the laboratory’s internal archive by the laboratory technician who advised the value. Laboratory technicians register information about the communication (the critical value, the technician who communicated the information and the person who received the information). The laboratory medical specialist also adds a comment to the critical result in the LIS, such as “critical value notified by phone”, so that this information is recorded in the patient’s clinical history.

The list of critical value limits used in our laboratory at the time of the study is presented in Table 1. In our laboratory, these limits were established based on literature sources, and were subsequently revised and approved by laboratory medical specialists and clinicians (*19*). In some cases, special critical values (serum calcium for intensive care unit (ICU) patients or potassium for nephrology outpatients) have been adopted. These critical values were derived from agreements with medical services. For patients in the ICU, a calcium lower than 1.75 mmol/L is not considered a critical value unless the serum albumin concentration is higher than 25 g/L. On the another hand, if the outpatient belongs to the Nephrology Service, a serum potassium concentration up to 6.2 mmol/L is considered a critical value, but is not necessary to report under an agreement between the Nephrology Service and our laboratory. Re-evaluation of these critical values is performed together with the clinicians when the need to modify a critical value arises, such as following the publication of new data, new accords with clinical services, or technological improvements.

**Table 1:** Critical values limits list for biochemical and haematological parameters

**Parameters**	**Lower critical values limit**	**Upper critical values limit**
P-Calcium (II), mmol/L	< 1.75	> 3.20
P-Calcium ion, mmol/L	< 0.85	> 1.60
Gas(B)-Carbon dioxide, mmHg	< 20	> 70
Hb(B)-Carboxihaemoglobin, %	-	> 10
B-Erythrocytes (Haematocrit)	< 0.15	-
P-Glucose, mmol/L	< 2.5	> 27
B-Haemoglobin, g/L	< 50	-
Gas(B)-Oxygen, mmHg	< 40	-
P-Phosphate, mmol/L	< 0.39	-
Pt (B)-Plasma; pH	< 7.2	> 7.6
P-Potassium ion, mmol/L	< 2.8	> 6.2
P-Sodium ion, mmol/L	< 120	> 160
B-Thrombocytes, x10^9^/L	< 30	-
B – blood. Hb – haemoglobin. P – plasma. Pt – patient.		

For this study, we defined critical values as those which exceed the critical value limits established for a single parameter for the same patient within a 24-hour period.

### Statistical analysis

The percentage of critical values was calculated as: total number of critical values detected for each parameter (N critical values)/number of test results reported for each parameter (N test results) × 100. The percentage of critical values relative to all critical values was calculated as: N critical values/total number of critical values × 100.

For this study, the patient’s results were separated into two groups: STAT laboratory test results and routine laboratory results. The routine laboratory results were further subdivided into inpatients and outpatients.

The percentage of critical values notified for emergency patients, inpatients and outpatients were calculated for each parameter as: number of critical values notified (N critical values notified) / total number of critical values (N critical values) × 100.

All data were calculated using Microsoft Excel (Microsoft Office 2003®, Alburquerque, EEUU).

## Results

During the 6-month study period, our clinical laboratory performed more than 1.4 million routine tests and approximately half a million STAT tests. In the same period, the number of critical values detected was 5723 (0.4%), approximately 80% of which were from STAT testing (4577), 15% from routine inpatient testing (884) and 5% from routine outpatient testing (262). The prevalence of critical values was 0.9% for STAT testing and 0.08% for routine testing. The frequency of critical values was calculated for each parameter, as shown in Table 2. The parameter with the highest percentage of all critical values was oxygen partial pressure (17.7%), followed by potassium ion (17.6%), thrombocytes (14%), carbon dioxide partial pressure (13.2%), calcium (II) (12.3%) and pH (11.2%).

**Table 2:** Parameters and number of test results reported for each parameter

**Parameters**	**N test results**	**N critical values**	**% critical values**	**% of all critical values**
Gas(B)-Oxygen	22,223	1011	4.5	17.7
P-Potassium ion	118,386	1007	0.9	17.6
B-Thrombocytes	112,015	802	0.7	14
Gas(B)-Carbon dioxide	44,144	756	3.4	13.2
P-Calcium (II)	44,420	703	1.6	12.3
Pt (B)-Plasma; pH	44,168	642	2.9	11.2
P-Glucose	122,836	337	0.3	5.9
P-Sodium ion	115,925	206	0.2	3.6
B-Haemoglobin	112,958	104	0.1	1.8
B-Erythrocytes (haematocrit)	112,141	80	0.1	1.4
P-Calcium ion	3144	40	1.3	0.7
P-Phosphate	19,249	34	0.2	0.6
Hb(B)-Carboxihaemoglobin	25	1	4.0	0
B – blood. Hb – haemoglobin. P – plasma. Pt – patient. N test results – number of total test results. N critical values - total number of critical values detected. % critical values - percentages of critical values with respect to number of test results for each parameter. % of all critical values - percentages of critical values with respect to all critical values.				

Tables 3 and 4 show the number of critical values, the number of critical values notified and the percentage of notified critical values observed for STAT tests, inpatients and outpatients for each parameter. For STAT laboratory tests, the majority of parameters resulting in critical values belonged to the patients in the emergency department. For routine laboratory tests, the highest percentage of critical values was found for inpatients in the ICU.

**Table 3:** Percentages of critical values notification for STAT patients

	**STAT TESTING**		
**Parameters**	**N critical values**	**N critical values notified**	**% critical values notification**
Gas(B)-Oxygen	1011	40	4
Gas(B)-Carbon dioxide	756	102	13.5
P-Potassium ion	652	418	64.1
Pt (B)-Plasma; pH	642	447	69.6
B-Thrombocytes	538	210	39.0
P-Calcium (II)	420	167	39.8
P-Glucose	229	157	68.6
P-Sodium ion	168	71	42.3
B-Haemoglobin	91	55	60.4
B-Erythrocytes (haematocrit)	69	48	69.6
Hb(B)-Carboxihaemoglobin	1	0	0
B – blood. Hb – haemoglobin. P – plasma. Pt – patient. N critical values - total number of critical values detected. N critical values notified - number of critical values notified. % critical values notification - percentage of critical values notification.			

**Table 4:** Percentages of critical values notification for inpatients and outpatients

	**INPATIENTS** **LABORATORY TESTING**			**OUTPATIENTS** **LABORATORY TESTING**		
**Parameters**	**N critical values**	**N critical values notified**	**% Critical** **values** **notification**	**N critical values**	**N critical values notified**	**% Critical** **values notification**
P-Potassium ion	242	194	80	113	70	62
B-Thrombocytes	209	4	2	55	2	4
P-Calcium (II)	285	166	58	38	25	66
P-Glucose	74	68	92	34	20	59
P-Sodium ion	21	17	81	17	4	24
B-Haemoglobin	11	9	82	2	1	50
B-Erythrocytes (haematocrit)	9	7	78	2	1	50
P- Phosphate	33	30	91	1	0	0
B – blood. Hb – haemoglobin. P – plasma. Pt – patient. N critical values - total number of critical values detected. N critical values notified - number of critical values notified. % critical values notification - percentage of critical values notification.						

The parameters with the highest notification percentage for critical values in STAT laboratory tests were pH (69.6%), haematocrit (69.6%), glucose (68.6%), potassium ion (64.1%) and haemoglobin (60.4%). For inpatients, the parameters with the most notifications were glucose (91.9%), phosphate (90.9%), haemoglobin (81.8%), sodium ion (81%) and potassium ion (80.2%). In outpatients they were calcium (65.8%) and potassium (61.9%).

## Discussion

In this study, we analysed all data collected over a 6-month period to obtain an overview of how our system works with regard to the detection and reporting of critical values. On reviewing the literature, only few studies on critical value notification have been performed in our country, and our results are in accordance with published data (*20*). Comparing the obtained percentages of critical values in our laboratory with results published in the literature, the percentages for almost all parameters were similar (*21*-*23*). As in our study, the vast majority of critical results are related to patients in the ICU and emergency department (*17*). The highest proportion of critical values corresponded to oxygen partial pressure (17.7%), followed by potassium ion concentration (17.6%). The literature shows that potassium, sodium and glucose are responsible for the majority of critical results (*23*).

In STAT laboratory and routine laboratory tests for inpatients, the parameters with the highest percentage of critical value reporting are pH, haemoglobin, glucose and potassium ion concentrations (*20*). In our study, haematocrit, phosphate and sodium ion concentrations were found to have the same notification percentages. For outpatients, calcium and potassium were the parameters with the highest proportion of critical value notifications. In the literature calcium and potassium showed lower levels of notification (*19*, *20*).

For serum calcium concentration, our findings for inpatients in the ICU differed from those reported in the literature (*24*). These differences are due to an agreement with clinicians at our hospital, whereby, it is not necessary to notify clinicians if the serum calcium concentration is lower than 1.75 mmol/L unless the patient’s serum albumin concentration is higher than 25 g/L.

When routine laboratory tests were sorted by inpatients and outpatients, the critical value notification percentage was significantly higher for inpatients than for outpatients (except for thrombocytes and calcium). This indicates that the notification system for outpatients is weaker, and more effort should be made to improve it. Other studies have also shown a lower notification percentages for outpatients (*15*).

Laboratory staff should be educated about the results and learn the entire list of critical values, reinforcing the importance of communicating all of them. An indicator of the quality of this process may be the critical value reporting rate, as the failure to report these values, estimated at 0.1% to 10%, could be indicative of the operational efficiency of laboratories (*14*). Different studies have demonstrated that the notification of critical values varies between tests, with percentages varying from 0.7% to 9%, similar to that found in our study (*15*).

This study has been useful in enabling us to observe the critical value notification percentage, and we were able to detect flaws in the annotation system of the communication process. Indeed, these values were reported to clinicians, but were not always registered in the laboratory’s internal archive. These values are initially communicated to clinicians, and once they are advised a comment is registered in the LIS. This second step was forgotten in several cases. This explains why the communication percentage was much lower than expected. Due to the obtained results, a reminder was made to all laboratory staff to ensure this second step is carried out.

Most guidelines demonstrate that direct calling is still the most used method for communicating critical values and, as such, all communication should be fully documented. Recent studies compared automated alert systems to telephone notifications from a call centre (*17*). Automated notification systems are automated alert systems or computerised reminders *via* mobile phones, pagers, email or other personal electronic devices, used to alert clinicians of critical value laboratory test results (*14*, *25*-*27*). Upon receipt of an automated notification, the responsible personnel acknowledge the critical value and confirm receipt of the alert. If the alert is not acknowledged within a specified timeframe, these systems typically revert to a manual notification system. Findings of previous studies have demonstrated better timing of reports for automated alerts than traditional systems. Another important issue is who should receive the result. In some studies, the vast majority of clinical laboratories consider the appropriate personnel to receive the critical value notification to be the physician who requested the test, any physician responsible for the patient or the nurse responsible for the patient (*28*).

Our study is one of few studies performed in a tertiary hospital, which also employs different methods of critical value notification. As two main points, the obtained results demonstrate the importance of different critical value definitions for special situations (*e.g.,* serum calcium for ICU patients), as well as the importance of registration of critical value communication. It is necessary to incorporate actions to improve not only critical value notification, but especially the registration of this activity. Therefore, we believe this study will be of interest to other clinical laboratories, and recommend that they undertake this kind of study as it highlights the weaknesses and strengths of a system. This study shows that notification system for outpatients is weaker than for inpatients. In another hand, we detected that most of the critical values although they were communicated, it were not registered in laboratory LIS. This evaluation helps us to educate to all laboratory staff in order to register all notifications.

A limitation of our study is that it was conducted retrospectively, and only evaluates data collected during a 6-month period. A prospective study could be designed to verify the obtained data and evaluate the results after improvement of the process. Another limitation is that we did not analyse the time between receipt of the samples and communication of the critical values, which would be an interesting aspect to investigate in future studies.
